# Psychometric validation of the Taiwanese nurse burnout scale

**DOI:** 10.1186/s40359-026-04040-4

**Published:** 2026-01-28

**Authors:** Meng-Ting Chen, Yi-Wen Chang, Chung-Ying Lin, Yanhong Dong, Miaofen Yen, Huan-Fang Lee, Hsiang-Chin Hsu

**Affiliations:** 1 Department of Nursing, College of Medicine, National Cheng Kung University, Tainan, Taiwan; 2 Nursing Department, National Cheng Kung University Hospital, College of Medicine, National Cheng Kung University, Tainan, Taiwan; 3 Nursing Department, Chi Mei Medical Center, Yongkang District, Tainan, Taiwan; 4 Institute of Allied Health Sciences, National Cheng Kung University, Tainan, Taiwan; 5 School of Nursing, College of Nursing, Kaohsiung Medical University, Kaohsiung, Taiwan; 6 Alice Lee Centre for Nursing Studies (ALCNS), NUSMEDYong Loo Lin School of Medicine, National University of Singapore, Queenstown, Singapore; 7 School of MedicineCollege of Medicine, National Cheng Kung University, Tainan, Taiwan; 8 Department of Emergency Medicine, National Cheng Kung University Hospital, Tainan, Taiwan

**Keywords:** Reliability, Validity, Nurses, Burnout, Psychometric properties

## Abstract

**Background:**

Nurse burnout has a negative impact on nurse retention, well-being, and the quality of patient care. The differences in healthcare systems and cultures also lead to the differences in perceptions of nurses burnout. The aim of this study was to evaluate the validity and reliability of the nurse burnout scale, a context-specific instrument for Taiwanese nurses.

**Methods:**

This methodological study was conducted in three medical centers across Taiwan using secondary data from a National Science Council project. The Taiwan Nurse Burnout Scale (TNBS) was developed through literature review, qualitative interviews, expert validation, and pilot testing. Psychometric evaluation included exploratory and confirmatory factor analyses to assess construct validity, and reliability. Data were analyzed using SPSS and JASP to ensure the scale’s validity and reliability for assessing burnout among Taiwanese clinical nurses.

**Results:**

The sample size of the final analytic sample was 529 nurses who were recruited in three medical Centers in Taiwan. A nine-factor and 42-item version of the Taiwanese Nurse Burnout Scale (TNBS) did not demonstrate the best fit indices in the confirmatory analysis, which led to the accounting of an alternative structural model. Exploratory factor analysis (EFA) of the revised scale resulted in a five-factor, 19-item solution that explained 73.2 per cent. of the total variance. A following confirmatory factor analysis (CFA) indicated acceptable fit, where the standardized loadings ranged between.58 to.95, average variance extracted (AVE) ranged between 0.58 and.85, the composite reliability was between.85 and. 94 and the Cronbach’s α 0.92 of the entire scale, thus validating the construct validity and reliability of the instrument.

**Conclusions:**

The five-dimensional, 19-item Taiwan Nurse Burnout Scale (TNBS) is indicated to have a strong psychometric face, thus making it an effective and reliable tool in the evaluation of burnout among Taiwanese clinical nursing groups.

**Supplementary Information:**

The online version contains supplementary material available at 10.1186/s40359-026-04040-4.

## Introduction

### Nurse burnout as global and context-sensitive challenge

Nurse burnout is a critical and persistent challenge in healthcare systems and a major contributor to nursing staff turnover [1, 2]. Accumulating evidence indicates that burnout is associated with compromised patient safety and quality of care, lower patient satisfaction, and diminished nurses’ organizational commitment and productivity [3, 4]. Accordingly, healthcare organizations increasingly recognize the need to foster a psychologically safe and emotionally supportive work environment to sustain nurses’ well-being and care delivery [5].

Cross-cultural nursing research advances understanding of both universal aspects of nurses’ experiences and context-specific factors shaping nursing practice and workforce outcomes [4, 6]. However, existing cross-national evidence has predominantly focused on Western countries, particularly the United States and selected countries of Europe [4, 7]. Studies have found that the prevalence of nursing burnout in Western countries is approximately 34% in the United States [8, 9].In Europe, a multi-country study involving 67 acute care hospitals across Belgium, England, Germany, Ireland, Norway, and Sweden reported that burnout prevalence ranged from 27% to more than 30%, with differences between countries and clinical settings. Nurses working in Germany, Ireland, Scandinavian countries, and England exhibited higher burnout rates compared with nurses in Belgium [3, 4, 8].

Research consistently found that adverse job characteristics—high workload, time pressure, negative nurse-physician relationship, poor supervisor/leader support, and negative team relationship—were associated with burnout in nursing [3, 4, 10]. Importantly, these structural and interpersonal conditions differ substantially across healthcare systems, which may explain why burnout prevalence varies across countries. In Eastern countries, prevalence levels appear similarly, with studies in Japan, and Thailand indicating rates of around 30–40% among clinical nurses [4, 11, 12]. In Taiwan, burnout appears particularly prevalent, with a national survey reporting that nearly 79% of the nurses experienced moderate to severe burnout [13]. Collectively, these findings indicate that nurse burnout is widespread global concern and this phenomenon adversely impacts their well-being, relationships, and job performance [2, 14, 15]. Furthermore, nurses’ burnout increases the risk of medical errors and hospital-acquired infections, ultimately compromising patient safety and diminishing the quality of care [16, 17].

### Limitations of existing burnout measures in nursing contexts

Ensuring the psychometric properties of measurement instruments on burnout is essential for accurately assessing burnout in practical, real-world clinical contexts [18]. The most commonly used tools for measuring burnout are the Maslach Burnout Inventory-Human Services Survey (MBI-HSS), developed by Maslach and Jackson [19], and the Copenhagen Burnout Inventory (CBI), created by Kristensen et al. [20]. Despite their widespread adoption, these instruments present notable limitations when applied to nursing populations in non-Western contexts.

The original MBI-HSS was developed in Western service-oriented professions and therefore does not fully reflect the professional demands of nursing. Moreover, because its items were shaped by Western assumptions about emotional expression and interpersonal work dynamics, the instrument fails to capture cultural burnout manifestations found in Asian nursing contexts. Additionally, the CBI consists of 19 items and was initially developed for general occupational burnout assessment but has been utilized in fields such as information technology and the oil industry [20]. Previous studies have indicated that the severity and manifestation of burnout may vary across cultural norms and healthcare system structures, yet it remains a serious global phenomenon [6, 8, 19]. According to a recent large-scale survey of 4,317 nurses, 37.1% were at high risk of burnout and 26.9% were at moderate risk [21]. A review of global nurse burnout research over the past 20 years showed a steady increase in publications and reported burnout among nurses in up to 110 countries, highlighting the challenge for healthcare managers to identify early signs and implement timely prevention and intervention measures [22].

### Rationale for developing Taiwanese nurse burnout scale

Cross-national studies suggest that differences in work environments, healthcare policies, and data collection protocols may compromise the cross-cultural validity of standardized instruments, such as the MBI-HSS [6, 23]. For example, in South Africa, contextual stressors—such as crime and threats to personal safety—substantially contribute to nurse burnout [24, 25]. In the United States and Canada, high nurse burnout rates have been linked to shorter average hospital stays [8, 26]. Notably, Japan deviates from this pattern: despite longer hospital stays, nurse burnout remains disproportionately high [6, 27, 28].

Taken together, these findings highlight the need for a nursing-specific, culturally grounded burnout assessment tools that reflects the realities of clinical nursing practice in Taiwan. Developing such an instrument would enhance both research precision and the design of targeted interventions, enabling healthcare organizations to better identify burnout risk factors and implement context-sensitive strategies. Accordingly, this study aimed to develop and psychometrically validate the Taiwanese Nurse Burnout Scale (TNBS), a context-specific instrument designed to assess burnout among Taiwanese clinical nurses.

## Methods

### Study design

This research was a methodological study in Taiwan.

### Study samples and setting

This study employs secondary data analysis from the National Science Council project and is conducted at three medical centers located in Northern, Central, and Southern Taiwan. The pilot test included 30 nurses who had been employed for at least six months at a medical center in southern Taiwan. For the formal scale survey, the inclusion criteria required that nurses have at least six months of employment. The exclusion criteria included those currently on extended leave (such as maternity leave, sick leave, bereavement leave, or personal leave) exceeding one month, as well as those who had previously participated in the pilot test to avoid duplicate sampling. Convenience sampling was utilized for participant recruitment, and data collection at each medical center took approximately one month to complete.

### Scale development process

**Fig. 1:**
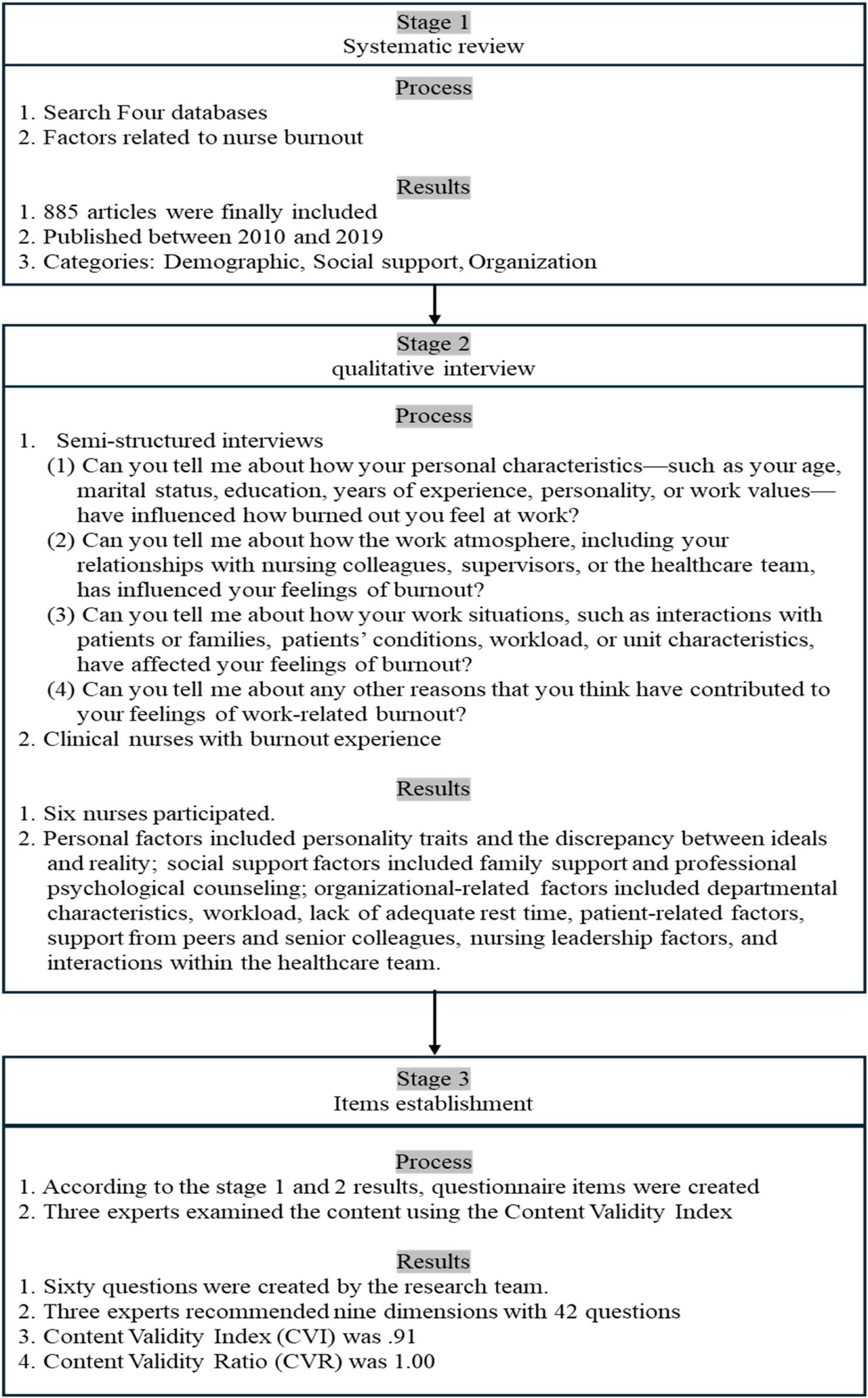
Each step of the initial scale development process

The original scale has been created in a three-stage process (Fig. 1). First, a systematic literature review was performed and factors related to nurse burnout. A total of 9,547 articles were identified in four scholarly databases: Embase, MEDLINE, Cochrane Library, and Scopus. The deletion of duplicates left 5,600 records. This was further filtered to 1,484 articles in a second preliminary screening on thematic relevance. Finally, 885 studies with a year of publication between 2010 and 2019 were categorized based on the burnout-related themes, thus creating the corpus on which the thematic analysis is going to be conducted.

Second, qualitative interviews are used to explore further factors in the Taiwan healthcare situation. Semi-structured interviews, according to the literature review and discussion of the research team, were conducted with six participants who consisted of current employees and former employees, with the aid of four questions related to burnout, and those licensed to practice in Taiwan without being prohibited from having clinical practice experience and were free to express their burnout experiences by consenting to taped interviews. Nurses who had subordinate relationships with the researcher were eliminated. The data were collected in an empty conference room in a one-on-one semi-structured in-depth interview, which lasted not more than 60 min each. The participants had the option of requesting a second interview. Every session was taped and transcribed directly. They had clinical experience, less than two years and greater than ten years of experience with a wide range of work settings, including, but not limited to, intensive care units, emergency units, general wards, and hemodialysis units. NVivo software was used to code data, and content analysis was conducted in accordance with Liang et al. [29, 30]; the categories and themes have thus been determined. The analytical procedure, as well as the final report, was cross-validated with three Ph.D. scholars in the field of qualitative research and two nursing professors. The research participants reviewed and provided approval on all the findings of the interviews.

Third, based on the systematic literature review and the Taiwanese nurse interviews, the research team developed a nine-dimensional questionnaire with a 60-item questionnaire that summarized nine dimensions. The instrument was reviewed by three experts, providing recommendations after following two rounds of reviews. Lastly, the preliminary nine dimensions with a 42-item scale demonstrated an overall Content Validity Index (CVI) of 0.91. Each item was computed according to the methodology by Lawshe [31], with respect to the content validity ratio (CVR), which relied on the ratings of experts regarding the necessity of the item. Out of the original list of items, 35 items reached a Content Validity Ratio (CVR) of 1.00. Eighteen items with CVR values between − 0.33 and − 1.00 were removed. Fifteen items with a CVR of 0.33 were set to be modified further, seven of which remained after being re-evaluated by all experts. Finally, 42 items were included after all experts confirmed.

The Taiwanese Nurse Burnout Scale included the following dimensions: rest quality (4 items), working hours (4 items), workload (4 items), care recipients (4 items), workplace support (7 items), work ability (5 items), family and social relationships (5 items), self-accomplishment (5 items), and nursing identity (4 items). Responses to the Taiwanese Nurse Burnout Scale are measured using a 7-point Likert-type scale, ranging from 1 (‘Strongly Disagree’) to 7 (‘Strongly Agree’), with intermediate values indicating increasing levels of agreement: 2 (‘Disagree’), 3 (‘Somewhat Disagree’), 4 (‘Neutral’), 5 (‘Somewhat Agree’), and 6 (‘Agree’). The scale was specifically developed for this study and has not been previously published elsewhere (see supplementary material).

### Pilot test

To evaluate the appropriateness of the Taiwanese Nurse Burnout Scale, three experts with expertise in nursing burnout and mental health were invited. Each item was assessed for importance, relevance, and clarity using a 7-point rating scale, where higher scores indicated stronger agreement. Experts also evaluated the alignment of items with the research objectives and conceptual framework, offering recommendations for revisions, additions, or deletions. A pilot test with 30 participants was conducted to evaluate internal consistency, producing a Cronbach’s α of 0.96. Given that a Cronbach’s α above 0.90 signifies excellent reliability, these results provide strong evidence of the scale’s high internal consistency.

This study will conduct a descriptive statistical analysis of the participants’ demographic data, examining variables such as gender, age, educational level, marital status, current unit, years of service within the institution, organizational level, professional rank, scheduled working hours, and average daily working hours.

### Data collection

The study was approved by the Institutional Review Board on April 26, 2019. The pilot testing of the scale was conducted from May to June 2020 at a medical center in southern Taiwan. The formal data collection of the scale was conducted from May to December 2020.

The pilot version of the scale was tested at a medical center. A stratified random sampling strategy was employed across various clinical units, including general medicine, surgical divisions, emergency services, and critical care units—resulting in 30 completed and valid responses. The final version of the scale was implemented at medical centers in Northern, Central, and Southern Taiwan.

### Data analysis

Data were analyzed using Microsoft Excel for preliminary organization and descriptive statistics, while inferential statistical procedures were conducted using SPSS version 17.0 and JASP software. To examine the demographic profile of the nursing personnel, descriptive statistical methods—specifically, the calculation of means and standard deviations—were employed.

Construct validity of the instrument was tested to confirm the suitability. There was extensive use of both discriminant and convergent validity processes to test construct validity comprehensively to ensure that the intended theoretical construct is not only effectively captured by the instrument used to measure it (through convergent validity) but also completely differentiated (through discriminant validity). Such a validation process determines the specificity and uniqueness of the measurement effects and thus proves that the scale measures the target construct and not other incidental attributes. Confirmatory factor analysis (CFA) and average variance extracted (AVE) were used to measure discriminant validity. Loadings based on CFA were used to differentiate between latent factors, but AVE measured the amount of variance that could be attributed to each construct. Convergent validity refers to the level to which various measures of a single latent construct are intertwined. It was assessed on average variance extracted (AVE) and composite reliability (CR), which are important measures of convergent and internal consistency, respectively. A value of an AVE above 0.50 suggests that the construct is capturing over 50% of the variance in the indicators, and a CR value above 0.70 reflects desirable internal consistency. The joint use of these indices defines the attainment of an acceptable convergence by a latent construct [32, 33].

Considering that the Taiwanese Nurse Burnout Scale is a newly developed instrument, it is unclear whether it aligns well with the theoretical framework used for its development. In this regard, the analytic plan was two-fold [34, 35]: First, to evaluate if the Taiwanese Nurse Burnout Scale structure fits the theoretical framework used for its development. Second, to explore and verify the factor structure of the Taiwanese Nurse Burnout Scale if its structure does not fit well with the theoretical framework used for its development. Following the analytic plan, the Taiwanese Nurse Burnout Scale was examined to check if it fits well with the nine-factor structure using confirmatory factor analysis (CFA) and the entire sample (*N* = 529). If not, the dataset would be randomly divided into two equal subsamples to explore and confirm another potential factor structure [33]. Exploratory factor analysis (EFA, *n* = 264) would then be carried out to identify a potential factor structure to further verify by another CFA (*n* = 265).

In EFA, we implemented Bartlett’s sphericity test and the Kaiser-Meyer-Olkin (KMO) measure. The optimal factor structure was determined through parallel analysis [33, 36]. The extraction process employed principal axis factoring with oblique rotation.

The process of item reduction was conducted in three stages. At stage 1, items were dropped when they were problematic in terms of cross-loading, which was operationally defined as a difference between loadings of less than 0.15. At stage 2, other items were dropped when they could not fit conceptually into the originally postulated factor structure as identified using a literature-informed theoretical review. At stage 3, the deletions and the set of retained items were reviewed and validated by all experts.

The subsequent CFA utilized maximum likelihood estimation with full information maximum likelihood (FIML) to address missing data, ensuring valid model parameters with positive error variances and standardized coefficients below 0.95 [33]. A comprehensive model assessment incorporated multiple fit indices: chi-square relative to degrees of freedom (χ²/df), comparative fit index (CFI), Tucker-Lewis index (TLI), root mean square error of approximation (RMSEA), and standardized root mean square residual (SRMR). The construct validity of the measurement model was rigorously evaluated through examinations of both convergent and discriminant validity according to established criteria [33]. Finally, internal consistency of the refined Taiwanese Nurse Burnout Scale was evaluated using Cronbach’s α [33].

### Ethical considerations

All procedures in the current study adhere to the Declaration of Helsinki. This study was also approved by the Institutional Review Board of the National Cheng Kung University Hospital (No. A-ER-108-111). Ethical approval for this investigation was obtained through a thorough review process, with formal authorization granted by the Institutional Review Board (IRB) of the primary institution. Additionally, supplementary endorsements were secured from all relevant ethical governance authorities before initiating any recruitment activities. Before data collection commenced, all participants received a comprehensive explanation outlining the study’s purpose and their rights, which was included in the scale to ensure informed consent. Participants were assured that all data would remain strictly confidential, with access limited solely to the research team, and that no personally identifiable information would be disclosed. All questionnaires were anonymized and coded for academic research purposes only. The act of completing and submitting the questionnaire was considered an indication of the participant’s informed consent to participate in the study.

## Results

### Sample

The final analytical cohort consisted of 529 nurses recruited from three geographically diverse tertiary care institutions located in northern, central, and southern Taiwan. The demographic analysis indicated a predominance of female practitioners, with the majority holding a bachelor’s degree. Professional experience varied significantly, ranging from early-career professionals with a minimum of one month of experience to highly seasoned practitioners with a maximum of 35 years of experience. The mean duration of clinical practice across the sample was 9.4 years. Most participants were employed in non-critical care settings (67.7%). Although the standard shift was officially set at 8 h (99.2%), actual working hours frequently exceeded this duration. Specifically, 56.0% of participants reported averaging 10-hour workdays, while 39.9% adhered to the standard 8-hour shifts.

### Item analysis

An item analysis of the Taiwanese Nurse Burnout Scale was conducted using mean, standard deviation, Cronbach’s α, and critical ratio (CR) values. The overall mean score was 3.23 (SD = 1.29), with item means ranging from 2.45 (SD = 1.22) to 4.41 (SD = 1.41). Corrected item-total correlations ranged from 0.44 to 0.71. The overall Cronbach’s α was 0.96, with minimal variation upon item deletion. Independent t-tests revealed significant differences between high- and low-score groups for all 42 items. As no item demonstrated poor psychometric properties, all were retained (Table 1). 

**Table 1:** Item analysis of the Taiwan nurse burnout scale

Item number	Mean	SD	Corrected Item-Total Correlation	Cronbach’s α if item deleted	CR^*^	Retention
1.	4.41	1.40	.49	.96	11.48	◎
2.	4.07	1.33	.63	.96	15.49	◎
3.	3.24	1.49	.44	.96	11.06	◎
4.	3.90	1.45	.54	.96	12.89	◎
5.	4.29	1.31	.63	.96	15.63	◎
6.	3.77	1.27	.71	.96	20.29	◎
7.	3.84	1.30	.69	.96	19.40	◎
8.	3.86	1.27	.62	.96	15.14	◎
9.	4.06	1.26	.64	.96	16.31	◎
10.	3.77	1.26	.67	.96	18.13	◎
11.	3.31	1.36	.68	.96	20.11	◎
12.	3.57	1.36	.62	.96	16.41	◎
13.	2.78	1.26	.62	.96	16.63	◎
14.	3.21	1.25	.64	.96	17.29	◎
15.	3.64	1.37	.59	.96	16.12	◎
16.	2.84	1.13	.61	.96	18.41	◎
17.	2.98	1.17	.66	.96	19.00	◎
18.	3.12	1.29	.66	.96	18.67	◎
19.	2.69	1.22	.58	.96	16.34	◎
20.	3.02	1.35	.60	.96	16.79	◎
21.	3.10	1.23	.67	.96	20.20	◎
22.	2.82	1.16	.59	.96	17.13	◎
23.	3.17	1.35	.70	.96	23.55	◎
24.	3.91	1.44	.57	.96	14.51	◎
25.	3.33	1.23	.66	.96	18.64	◎
26.	3.06	1.20	.62	.96	17.25	◎
27.	2.90	1.14	.64	.96	17.80	◎
28.	2.87	1.13	.63	.96	17.60	◎
29.	2.44	1.21	.54	.96	15.11	◎
30.	2.55	1.21	.61	.96	18.30	◎
31.	2.74	1.26	.63	.96	18.11	◎
32.	2.65	1.22	.61	.96	16.15	◎
33.	2.92	1.45	.58	.96	15.57	◎
34.	3.33	1.34	.67	.96	18.35	◎
35.	3.05	1.23	.71	.96	21.93	◎
36.	2.93	1.24	.64	.96	19.55	◎
37.	2.98	1.18	.68	.96	20.50	◎
38.	3.14	1.29	.67	.96	20.14	◎
39.	2.91	1.32	.66	.96	20.39	◎
40.	2.69	1.16	.59	.96	15.92	◎
41.	2.72	1.29	.63	.96	19.43	◎
42.	3.09	1.40	.60	.96	16.41	◎

* *p* < .001 for all Critical Ratio, ◎: Retain this item, Cronbach’s α for the total scale = .96

### Confirmatory factor analysis (original model)

Based on the theoretical framework of the Taiwanese Nurse Burnout Scale, a hypothetical measurement model was established with nine dimensions and 42 items. The overall model fit was tested using maximum likelihood estimation, yielding the following fit indices: χ² (*p* < .001), χ²/df = 4.90, GFI = 0.84, SRMR = 0.07, RMSEA = 0.08, TLI = 0.81, and CFI = 0.83. These results indicate that the model exhibited poor fit.

### Exploratory factor analysis

**Fig. 2:**
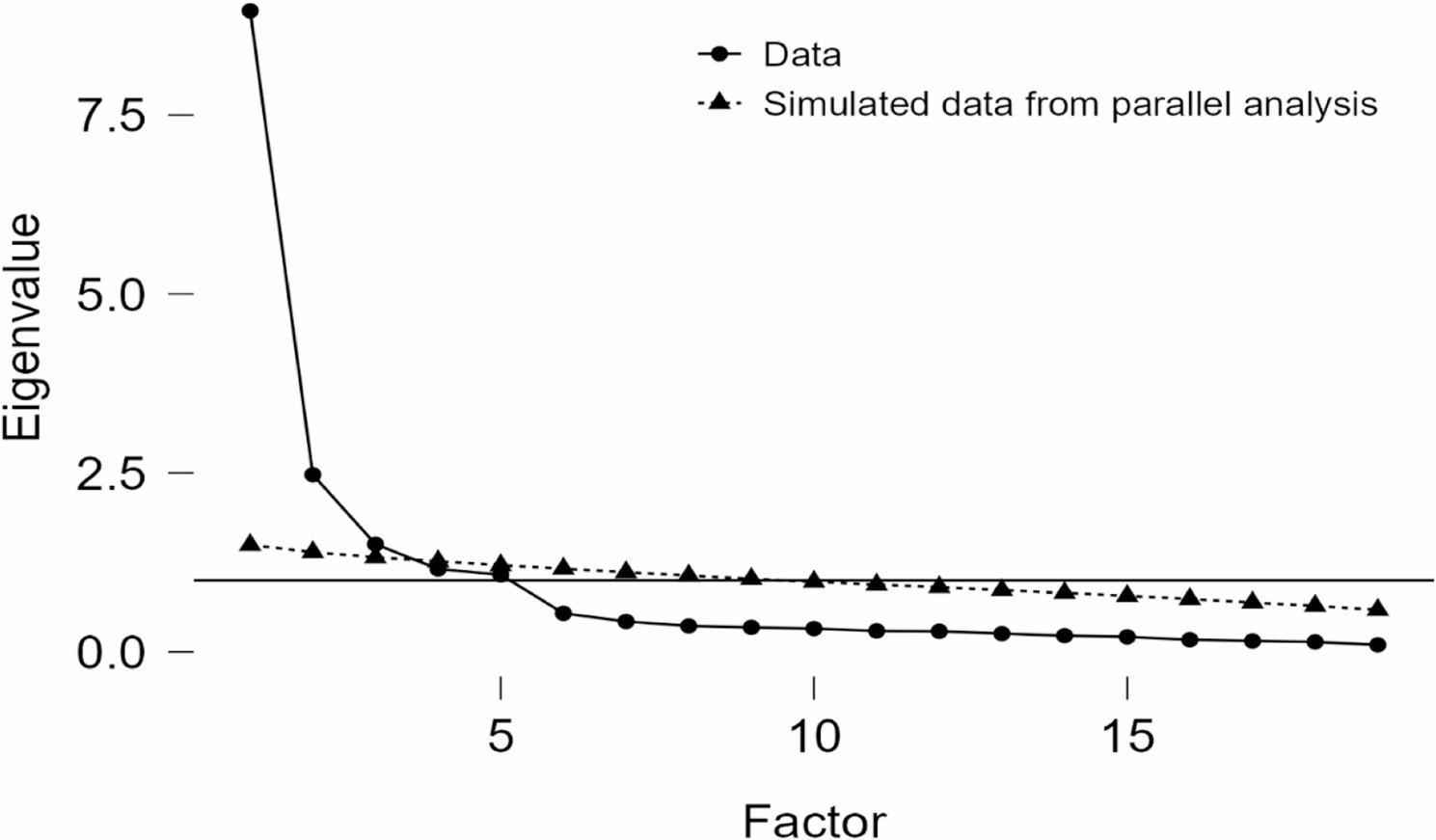
Scree plot

EFA was applied to the 42-item Taiwanese Nurse Burnout Scale based on data collected from a sample of 264 participants. The suitability of the dataset for factor analysis was supported by the KMO measure of sampling adequacy, which yielded a value of 0.94, reflecting an excellent level as suggested by Kaiser [36]. Furthermore, Bartlett’s test produced a statistically significant result (χ² = 10079.20, *p* < .001), indicating that the correlation matrix was not an identity matrix and thus factor analysis was appropriate. Factor selection was determined using parallel analysis. To facilitate data reduction, principal components analysis was initially performed, identifying five factors for extraction (Fig. 2). Subsequently, principal axis factoring with oblique rotation was applied, retaining five factors. The final model explained 73.2% of the total variance. During the first EFA, items with communalities below 0.4 and cross-loadings were removed. In the second EFA, a theoretical review based on existing literature indicated that certain items did not align with the originally hypothesized factor structure. All removed items and the final retained set were then reviewed by an expert panel. After further item elimination, the final scale comprised 19 items.

Factor loadings were examined to define the factor structure, with factors ranked according to their proportion of explained variance. The first factor, workload, comprising six items, explained 21.3% of the total variance, indicating its significant contribution to the overall construct. The second factor, workplace support, consisting of four items, explained 14.0% of the variance, followed by work ability (three items, 14.0%), nursing identity (three items, 12.2%), and family relationships (three items, 11.7%) (Table 2).

**Table 2:** Exploratory factor analysis of Taiwan version of burnout

Item number	Factor 1	Factor 2	Factor 3	Factor 4	Factor 5
Factor 1: workload					
9. My workload is so heavy that I feel mentally exhausted	.96				
10. The excessive nursing workload makes me feel on the verge of collapse	.84				
5. Day after day of nursing work makes me mentally fatigued	.84				
8. I feel that the long nursing working hours make me feel overburdened	.83				
6. Thinking about starting a new workday makes me feel very distressed	.72				
1. Breaks during work cannot relieve my fatigue	.67				
Factor 2: workplace support					
22. Interactions in my unit make me feel stressed		.87			
20. Interactions with my nursing supervisor cause me great stress		.83			
19. Interactions with fellow nurses cause me great stress		.83			
21. Interactions with the medical team cause me great stress		.65			
Factor 3: work ability					
26. I feel that my lack of ability prevents me from providing better nursing care			.95		
28. I feel that my lack of ability prevents me from coping with current nursing work			.92		
27. I feel that my lack of ability makes me lose confidence in participating in team interactions			.86		
Factor 4: nursing identity					
39. Nursing work feels meaningless to me				.90	
41. I do not want to continue my nursing career				.86	
40. I do not care whether I need to further improve my nursing skills				.79	
Factor 5: family relationships					
32. After a shift, interactions with others do not relieve my work stress					.90
31. After a shift, my stress has not been properly managed					.84
30. When no one understands my work stress, I feel helpless					.75
Initial Eigenvalues	8.95	2.47	1.50	1.15	1.07
Percentage of Variance Explained	.21	.14	.14	.12	.11
Cumulative % of Variance Explained	.21	.35	.49	.61	.73

### Confirmatory factor analysis (final model)

A new model was developed using EFA with the second sample, followed by CFA for validation. The sample comprised 265 nurses. The overall model fit indices were as follows: χ² (*p* < .001), χ²/df = 2.13, GFI = 0.96, SRMR = 0.05, RMSEA = 0.06, TLI = 0.94, and CFI = 0.95. Since χ² is sensitive to sample size, additional fit indices were taken into account. The results indicate that the proposed CFA model demonstrated a good fit with the observed data (Table 3).

**Table 3:** Fit indices of the model in confirmatory factor analysis

Fit indices	Good fit	Model	Fit status
*χ* ^*2*^	*p*> .050	*p* < .001	Poor fit
*χ* ^*2*^ */df*	<3.00	2.13	Good fit
GFI	>0.90	0.96	Good fit
SRMR	<0.08	0.05	Good fit
RMSEA	<0.08	0.06	Good fit
TLI	>0.90	0.94	Good fit
CFI	>0.90	0.95	Good fit

The standardized factor loadings for the 19 items ranged from 0.58 to 0.95, with all values exceeding 0.50 and demonstrating statistical significance.

The AVE for all five dimensions ranged from 0.58 to 0.85, surpassing the 0.50 threshold. The composite reliability (CR) values for all five dimensions ranged from 0.85 to 0.94, exceeding the 0.60 benchmark. These results confirm strong convergent validity.

Regarding discriminant validity, the square root of the average variance extracted for workload, workplace support, family relationships, work ability, and nursing identity exceeded the correlation coefficients between each factor and all other factors. These findings confirm that the 19-item Taiwanese Nurse Burnout Scale demonstrates strong discriminant validity.

### Reliability/internal consistency

To assess internal consistency, Cronbach’s α was calculated for the five dimensions. The overall Cronbach’s α for the 19-item scale was 0.92, indicating excellent reliability. The Cronbach’s α values for each dimension were as follows: workload = 0.90, workplace support = 0.85, family relationships = 0.88, work ability = 0.95, and nursing identity = 0.88.

## Discussion

This study provides empirical evidence that nurse burnout among Taiwanese medical center nurses is structured around a smaller number of core dimensions than initially hypothesized. Rather than supporting the original nine-dimension model, the refined five-factor structure suggests that nurses in high-intensity clinical settings tend to experience burnout through integrated and overlapping domains, particularly workload, workplace support, work ability, nursing identity, and family relationships. This consolidation reflects how burnout is perceived and experienced in practice, where multiple stressors are often intertwined rather than compartmentalized into distinct categories. The emergence of workload as the factor explaining the largest proportion of variance underscores its central role in shaping burnout experiences in tertiary hospitals, where high patient acuity, time pressure, and staffing constraints are pervasive. Similarly, workplace support formed a coherent and independent dimension, highlighting the importance of interpersonal relationships, leadership, and team dynamics as critical buffers against burnout. Together, these findings suggest that contemporary nurse burnout is less about isolated stressors and more about the cumulative impact of organizational demands and relational contexts, reinforcing the need for assessment tools that reflect real-world clinical complexity.

Burnout is a multifactorial construct, and prior studies have shown that existing models, including the MBI-HSS, exhibit limited explanatory power for its predictors [20, 37]. To address these limitations, the Taiwanese Nurse Burnout Scale was developed through systematic literature review and qualitative interviews with Taiwanese nurses to identify more precise burnout factors. The Taiwanese Nurse Burnout Scale is reliable and effective in assessing nurse burnout, as demonstrated by these findings. The MBI-HSS comprising emotional exhaustion, depersonalization, and personal accomplishment, has been extensively utilized in large representative samples across eight countries for cross-cultural validation. Findings have shown stable and consistent intercorrelations among the three dimensions in most countries, indicating that higher levels of emotional exhaustion or depersonalization are typically associated with lower personal accomplishment among nurses [6]. However, research conducted in Japan reported relatively weak or negligible correlations among these dimensions, suggesting that cultural factors may influence the psychological structure of burnout experiences [27].

The five-factor structure identified in this study can be meaningfully situated within established burnout theories, particularly the Maslach model, which conceptualizes burnout as comprising emotional exhaustion, depersonalization, and reduced personal accomplishment. In the present findings, workload and workplace support closely align with emotional exhaustion, reflecting sustained depletion of emotional and physical resources under excessive demands and limited organizational support. Work ability and nursing identity correspond conceptually to reduced personal accomplishment, capturing nurses’ perceived competence, professional efficacy, and sense of meaning in clinical practice. Although depersonalization did not emerge as a distinct factor, elements of emotional distancing and interpersonal disengagement appear to be embedded within the family relationships and workplace support dimensions, suggesting that relational strain may be experienced in a more contextually integrated manner in Taiwanese clinical settings. Rather than contradicting the Maslach framework, this consolidation indicates that classical burnout components may manifest through fewer, practice-oriented dimensions shaped by healthcare system characteristics and professional socialization. Thus, the Taiwanese Nurse Burnout Scale extends existing burnout theory by offering a context-sensitive operationalization that bridges foundational models with contemporary nursing practice.

Beyond a simplistic East–West dichotomy, recent cross-cultural research suggests that cultural differences in burnout operate through distinct behavioral routes shaped by organizational norms, learning processes, and emotion regulation strategies. Although nurses in both Eastern and Western contexts experience high levels of occupational stress, the ways in which stress is interpreted and translated into behavioral outcomes differ. For example, Frey et al. (2025) showed that nurses in Hong Kong reported higher burnout severity, whereas nurses in Switzerland exhibited stronger turnover intentions, indicating culturally distinct pathways linking stress exposure to disengagement and career decision-making.

At the organizational level, Western healthcare systems—often characterized by autonomy-oriented and innovation-driven cultures—tend to emphasize individual initiative, self-directed learning, and explicit emotional expression, whereas Eastern contexts more commonly prioritize relational harmony, role clarity, and implicit emotion regulation within hierarchical or clan-oriented structures [35]. A recent cross-cultural meta-analysis further demonstrated that clan and hierarchical cultures exert stronger individual-level effects in Eastern societies, while adhocracy and market-oriented cultures are more influential in Western contexts [35]. Together with evidence that psychological flexibility functions as a protective factor across cultures (Frey et al., 2025), these findings support interpreting burnout as one manifestation of culturally patterned adaptation to organizational demands rather than as a culture-specific outcome.

An important finding of this study is that the refinement from nine to five factors occurred not solely due to cultural influences but through empirical patterns observed in the dataset. Items representing “rest quality,” “working hours,” and “care recipients,” although theoretically meaningful, demonstrated low communalities or cross-loadings. These patterns suggest that nurses working in Taiwanese medical centers may no longer perceive these stressors as distinct domains but rather as embedded components of overall workload or workplace support. This interpretation aligns closely with the statistical structure rather than broad cultural assumptions.

For example, the strong loadings and high internal consistency of the workload and workplace support dimensions indicate that these two domains remain central pathways through which burnout manifests among Taiwanese nurses. The prominence of workload as the factor explaining the largest proportion of variance (21.3%) empirically reflects the organizational characteristics of tertiary hospitals, which are marked by high patient acuity, complex care coordination, and chronic staffing shortages. Similarly, workplace support emerged as a coherent construct that captures team communication, supervisory support, and interprofessional dynamics—elements historically identified as protective factors against burnout in high-intensity settings.

Generational value shifts, which we initially highlighted in cultural terms, are better interpreted as organizational and cohort effects observed in the psychometric data. Younger nurses’ responses displayed higher convergence within the “nursing identity” and “work ability” factors in the CFA model. This pattern suggests that nurses trained under recent competency-based curricula may conceptualize professional identity and clinical capability as integrated constructs rather than separate domains. These findings provide an empirically grounded explanation for why several items in the original nine-dimension model failed to form independent factors.

Rather than attributing measurement differences solely to collectivist or individualist cultural orientations, our data indicate that burnout experiences among Taiwanese nurses are shaped by a combination of institutional demands, evolving professional training, and cohort-specific expectations of the work environment. Thus, while cultural context remains relevant, the psychometric results suggest that organizational realities and generational transitions exert more direct influence on how burnout is structured and perceived. This interpretation advances a more nuanced and data-centered understanding of the scale’s factor refinement.

### Strengths and limitations of the study

This methodologically rigorous development process ensures the scale’s content validity and contextual relevance by aligning its constructs with both empirical findings and real-world nursing experiences. By integrating qualitative insights with quantitative validation, the TNBS captures the complexity of nurse burnout, addressing both individual experiences and systemic factors in a way that is culturally and professionally relevant.

Despite its robust methodological foundation and empirical validation, this study has certain limitations that warrant consideration. Firstly, this study employed secondary data analysis, which inherently limited control over variable selection, measurement reliability, and data completeness. Secondly, manpower and time constraints confined data collection to urban hospitals, excluding hospitals in rural and eastern regions. This limitation may hinder the generalizability of findings, as regional differences in working conditions, staffing levels, and resource availability can affect burnout patterns. Thirdly, this focus on medical center nurses excluded those working in regional hospitals, primary care clinics, public health centers, schools, and long-term care facilities. Since burnout is influenced by work environments, hospital hierarchies, and patient acuity levels, the exclusion of diverse healthcare settings restricts the generalizability of findings to the broader nursing workforce. Lastly, secondary data analysis precludes direct researcher control over data collection, limiting the ability to customize study variables, refine measures, address missing data, or clarify inconsistencies.

### Clinical implications

The validated 19-item, five-dimension TNBS offers several clinically meaningful implications that are directly grounded in the study’s psychometric findings. First, given that workload and workplace support emerged as the two factors explaining the largest proportion of variance, the TNBS can be used in clinical practice as a targeted screening tool to identify unit-specific burnout drivers. Nurse managers may apply subscale scores to distinguish whether burnout primarily stems from excessive workload demands or from insufficient interpersonal and organizational support, thereby enabling more precise and context-sensitive interventions.

Second, the strong reliability and construct validity of the TNBS support its use as a routine monitoring instrument within healthcare organizations. Regular assessment using the TNBS may facilitate early detection of burnout trends among nursing staff, allowing administrators to implement timely preventive strategies before burnout progresses to turnover, absenteeism, or compromised patient care. The refined scale’s brevity further enhances its feasibility for repeated use in busy clinical settings.

Third, the inclusion of “nursing identity” and “work ability” as empirically supported dimensions provides important implications for nursing education and professional development. Educational programs and in-service training may use TNBS results to design curricula or workshops that strengthen nurses’ professional role identity, clinical confidence, and perceived competence, particularly among early-career nurses. These findings highlight burnout not only as a stress-related outcome but also as a phenomenon closely linked to professional identity formation and perceived capability.

Finally, at the organizational and management level, TNBS data can inform workforce planning and policy development. Aggregated subscale profiles may guide resource allocation, such as staffing adjustments in high-workload units or leadership training initiatives aimed at improving workplace support. Moreover, the TNBS can serve as an outcome measure for evaluating the effectiveness of organizational interventions, such as workload redistribution, peer support programs, or mentoring systems, by comparing burnout levels before and after implementation.

## Conclusions

The Taiwanese Nurse Burnout Scale consists of 19 items that were created using a mixed-methods methodology to achieve cultural relevance and contextual validity. This measure is a sound and contextually suitable instrument for measuring burnout among Taiwanese nurses in future empirical research.

## Supplementary Information

Supplementary Material 1.

## Data Availability

The datasets used and analyzed during the current study are available from the corresponding author on reasonable request.
