# Supplementary material for: Psychometric validation of the Taiwanese nurse burnout scale
Source: BMC Psychol. 2026 Jan 28;14:465. doi: 10.1186/s40359-026-04040-4 (PMC13049859; doi:10.1186/s40359-026-04040-4)
Supplement: Supplementary file 1 — Supplementary Material 1. [file 40359_2026_4040_MOESM1_ESM.pdf]

42-item, nine-dimension Taiwanese Nurse Burnout Scale (TNBS)

Factor 1: quality of rest

1. Breaks during work cannot relieve my fatigue.
2. The fatigue from my nursing work has never improved.
3. I do not wish to spend extra time attending gatherings with family or friends.
4. I feel that I have never had a proper vacation.

Factor 2: working hours

5. Day after day of nursing work makes me mentally fatigued.
6. Thinking about starting a new workday makes me feel very distressed.
7. When I think about getting ready for work, I feel powerless.
8. I feel that the long nursing working hours make me feel overburdened.

Factor 3: work quantity

9. My workload is so heavy that I feel mentally exhausted.
10. The excessive nursing workload makes me feel on the verge of collapse.
11. I feel I am merely meeting unit requirements rather than actually caring for patients.
12. My workload is so heavy that I neglect patients' genuine care needs.

Factor 4: care recipients

13. Interactions with patients and family members are extremely distressing to me.
14. Caring for patients causes me considerable stress.
15. I often worry about sudden clinical deterioration and feel emotionally tense.
16. I feel insufficiently competent to care for the patients before me.

Factor 5: workplace support

17. Colleagues and senior nurses do not understand my work stress, and I feel helpless.
18. My supervisor does not understand my work stress, and I feel helpless.
19. Interactions with fellow nurses cause me great stress.
20. Interactions with my nursing supervisor cause me great stress.
21. Interactions with the medical team cause me great stress.
22. Interactions in my unit make me feel stressed.
23. When I am in the unit, I have no outlet to relieve my work stress.

Factor 6: work ability

24. When facing unstable or complex care situations, I feel emotionally tense.
25. When facing difficult care situations, I consistently feel unable to cope.
26. I feel that my lack of ability prevents me from providing better nursing care.
27. I feel that my lack of ability makes me lose confidence in participating in team interactions.
28. I feel that my lack of ability prevents me from coping with current nursing

work.

Factor 7: family relationships

29. My family does not provide support for my work stress, and I feel lonely.
30. When no one understands my work stress, I feel helpless.
31. After a shift, my stress has not been properly managed.
32. After a shift, interactions with others do not relieve my work stress.
33. I do not wish to spend time participating in various social gatherings.

Factor 8: professional accomplishment

34. I devote my nursing expertise without receiving adequate return.
35. I devote my nursing expertise but am not recognized by others.
36. I feel inferior compared with other members of the healthcare team.
37. My nursing competence has not been acknowledged.
38. My nursing professionalism is not respected.

Factor 9: nursing identity

39. Nursing work feels meaningless to me.
40. I do not care whether I need to further improve my nursing skills.
41. I do not want to continue my nursing career.
42. Doing any other occupation would be better than being a nurse.
